# New subclass of meromorphic harmonic functions defined by symmetric q-calculus and domain of Janowski functions

**DOI:** 10.1016/j.heliyon.2024.e38960

**Published:** 2024-10-09

**Authors:** Ahmad A. Abubakar, Khaled Matarneh, Suha B. Al-Shaikh, Mohammad Faisal Khan, Mustafa Kamal

**Affiliations:** a Faculty of Computer Studies, Arab Open University, Riyadh, 11681, Saudi Arabia; b Department of Basic Sciences, College of Science, and Theoretical Studies, Saudi Electronic University, Riyadh, 11673, Saudi Arabia

**Keywords:** 30C55, 30C45, Univalent functions, Symmetric *q*-calculus, Harmonic functions, Meromorphically harmonic functions, Janowski type function, Convolution, Meromorphically harmonic starlike functions

## Abstract

In this article, by applying the convolution principle and symmetric *q*-calculus, we develop a new generalized symmetric *q*-difference operator of convolution type, which is applicable in the domain $E^{⁎}=\left\{\tau :\tau \in C\text{ and }0<\left|\tau \right|<\infty \right\}$. Utilizing this operator, we construct, analyze, and evaluate two new sets of meromorphically harmonic functions in the Janowski domain. Furthermore, we investigate the convolution properties and necessary conditions for a function *F* to belong to the class $MS_{H}^{P,R}\left(q,q^{-1}\right)$, examining the sufficiency conditions for *F* to satisfy these properties. Moreover, we examine key geometric properties of the function *F* in the class $MS_{\bar{H}}^{P,R}\left(q,q^{-1}\right)$, including the distortion bound, convex combinations, the extreme point theorem, and weighted mean estimates.

## 1 Introduction and definitions

Suppose that A represent the set of analytic functions *h* inside the disk E={τ∈C:|τ|<1}. These functions are normalized so that h(0) is equal to 0 and $h^{{}^{\prime }}(0)$ is also equal to 1. A function F=u+iv, where *u* and *v* are real-valued harmonic functions (HFs) in *E*, is said to be harmonic in *E*. Furthermore, the harmonic function F=u+iv, which has complex values, may alternatively be represented as $F=h+\bar{g}$, where *h* and *g* are analytic functions inside the domain *E*. The set of such functions is represented by the symbol *H*. Specifically, the term *h* is used to denote the analytic component of harmonic functions *F*, where the term *g* is used to denote the co-analytic component of harmonic functions *F*.

The Jacobian of the harmonic functions is provided as:

$$J_{F}(\tau )={\left|h^{{}^{\prime }}(\tau )\right|}^{2}-{\left|g^{{}^{\prime }}(\tau )\right|}^{2}.$$

It is a well-established fact (see [1]) that any harmonic function $F=h+\bar{g}$ is locally univalent and sense preserving in *E* if and only if $J_{F}(\tau )>0$ in *E*, which is equal to $\omega (\tau )=\frac{g^{{}^{\prime }}(\tau )}{h^{{}^{\prime }}(\tau )}$ in *E*, subject to the condition that |ω(τ)|<1, for every τ∈E. For detail (see [2]).

Hengartner and Schober [3] proposed and investigated particular types of harmonic functions in $\overset{˜}{E}=\left\{\tau :\tau \in C\text{ and }\left|\tau \right|>1\right\}$ of the complex *τ*-plane C. In addition, they proved the existence of sense-preserving and univalency of harmonic functions *F*, which is defined in $\overset{˜}{E}$ and has the property F(∞)=∞, has to confirm to the following representation:

(1) $$F(\tau )=L_{1}(\tau )+L_{2}(\tau )+Q\log\left|\tau \right|$$

with Q∈C for $0\leq \left|\eta_{2}\right|<\left|\eta_{1}\right|$,

$$L_{1}(\tau )=\eta_{1}\tau +\sum_{t=2}^{\infty }a_{t}\tau^{-t}\;\;\text{and}\;\;L_{2}(\tau )=\eta_{2}\bar{\tau }+\sum_{t=1}^{\infty }b_{t}\bar{\tau^{-t}}.$$

Jahangiri and Silverman [4] established satisfactory coefficient criteria to determine the functions of the form (1) are univalent. In accordance with specific limitations, they also established criteria for coefficients that are both necessary and sufficient coefficient criteria to classify functions as harmonic and starlike [5]. Following this, Murugusundaramoorthy [6], [7] and Jahangiri [8] explored the groups of meromorphically harmonic functions in $\overset{˜}{E}$. In the following published articles [9], [10], Zou et al. introduced a technique for analyzing the meromorphically harmonic starlike functions in relation to symmetrical conjugate $E^{⁎}=\left\{\tau :\tau \in C\text{ and }0<\left|\tau \right|<\infty \right\}=E﹨\{0\}$.

In [10], a sharp estimation of the coefficient and a detailed characterization of these functions were also established. In order to enhance our comprehension of the fundamentals, we will use the symbol $M_{H}$ to represent the set of all meromorphic harmonic functions $F=h+\bar{g}$ that fulfill the normalized requirements h(0)=g(0)=0, $h^{{}^{\prime }}(0)=1$, and are in the form of

(2) $$F(\tau )=h(\tau )+\bar{g(\tau )}=\frac{1}{\tau }+\sum_{t=1}^{\infty }a_{t}\tau^{t}+\sum_{t=1}^{\infty }\bar{b_{t}\tau^{t}},\;\;\tau \in E^{⁎}$$

where h(τ) is analytic in $E^{⁎}$ and g(τ) is analytic in *E*, respectively, and may be expressed as series forms

(3) $$h(\tau )=\frac{1}{\tau }+\sum_{t=1}^{\infty }a_{t}\tau^{t},\;\tau \in E^{⁎}\text{ and}\;\;g(\tau )=\sum_{t=1}^{\infty }b_{t}\tau^{t},\;\left|b_{1}\right|<1,\;\tau \in E^{⁎}$$

and $a_{t}\geq 1$ and $b_{t}\geq 1$, for t≥2. Moreover, let $M_{\bar{H}}$ be a subclass of $M_{H}$ that consists of harmonic univalent functions of the type

(4) $$F(\tau )=\frac{1}{\tau }+\sum_{t=2}^{\infty }\left|a_{t}\right|\tau^{t}-\sum_{t=1}^{\infty }\left|b_{t}\right|\bar{\tau^{t}},\;\;\tau \in E^{⁎}.$$

Indeed, if g(τ)=0, then

(5) $$F(\tau )=g(\tau )=\frac{1}{\tau }+\sum_{t=1}^{\infty }a_{t}\tau^{t},\;\;\tau \in E^{⁎}$$

and

$$M_{H}=M,$$

where *M* is the class of meromorphic harmonic functions. Regarding the subject, we define the concepts of class of meromorphically harmonic starlike ($MS_{H}^{⁎})$ and the class of meromorphically harmonic convex functions ($MS_{H}^{C})$ in the domain $E^{⁎}$, which are defined as follows:

MSH⁎={F:F∈MH and Re(DHF(τ)F(τ))<0, τ∈E⁎}

and

MSHC={F:F∈MH and Re(DH(DHF(τ))DHF(τ))<0, τ∈E⁎}

where

$$D_{H}F(\tau )=\tau h^{{}^{\prime }}(\tau )-\bar{\tau g^{{}^{\prime }}(\tau )}.$$

The Hadmard (Convolution) of two functions analytic function $h_{1},h_{2}$ is defined as follows:

$$(h_{1}⁎h_{2})\tau =\sum_{t=1}^{\infty }a_{t}b_{t}\tau^{t},$$

where

$$h_{1}(\tau )=\sum_{t=1}^{\infty }a_{t}\tau^{t}\text{ and }h_{2}(\tau )=\sum_{t=1}^{\infty }b_{t}\tau^{t}.$$

Similarly, the Hadmard (Convolution) of two harmonic functions $F=h+\bar{g}$ and $F_{1}=h_{1}+\bar{g_{1}}$ is defined by the following way:

$$(F⁎F_{1})(\tau )=(h⁎h_{1})(\tau )+\bar{\left(g⁎g_{1})(\tau \right)}.$$

Now we will provide the concept of weak subordination in *E*. To get specific information on subordination for harmonic mappings, see [11], [12].

Definition 1 A complex valued function $F_{1}(\tau )$ in *E* is considered weakly subordinate to a function $F_{2}(\tau )$ in *E* if $F_{1}\left(\tau \right)⪯F_{2}\left(\tau \right)$, which is denoted as $F_{1}⪯F_{2}$. This relationship holds true if and only if $F_{1}(\infty )=F_{2}(\infty )$ and the image of $F_{1}(E)$ is a subset of the image of $F_{2}(E)$. Given that $F_{1}(\tau )⪯F_{2}(\tau )$ and $F_{2}$ is a univalent function in *E*, we may define a complex-valued function $w(\tau )=F_{2}^{-1}(F_{1}(\tau ))$, τ∈E. This function maps *E* onto itself and has the property that w(∞)=∞. Conversely, if the function $w(\tau )=F_{2}^{-1}(F_{1}(\tau ))$ in *E* and maps *E* onto itself, with w(∞)=∞, then $F_{1}⪯F_{2}$.

This may be stated in the following equivalent:

Lemma 1 *[13]* *. A complex valued function* $F_{1}$ *in E will be the weakly subordinate to another function* $F_{2}$ *in E, if and only if, with* w(∞)=∞ *, there exists a function w that maps E onto itself and* $F_{1}(\tau )=F_{2}(w(\tau ))$ *for every τ in E.*

In the nineteen century, numerous mathematicians applied *q*-calculus operator theory in diverse scientific fields, including fractional calculus, optimal control, *q*-integral equations, *q*-difference equations, and Geometric Function Theory (GFT). Jackson [14] introduced the *q*-derivative and *q*-integral operator in 1908 and looked at how they could be used. Based on the ideas of *q*-calculus theory, Ismail et al. [15] created the class of *q*-starlike functions in 1990, by implementing the concepts of *q*-difference operator. A number of scholars have recently conducted extensive research on *q*-calculus and GFT. The relevant works to refer to are as follows: [16], [17], [18], [19], [20], [21], [22], [23], [24], [25], [26], [27].

Currently, the exploration of the practical uses of symmetric *q*-calculus is a prominent subject in the realm of GFT. The significance of symmetric *q*-calculus is being shown in several domains, including fractional calculus and quantum physics [28], [29]. Sun et al. examined some of the properties of fractional *q*-symmetric integral and symmetric *q*-derivatives in their publication [30]. They used fractional difference operator and symmetric *q*-fractional integral to look at problems with non-local boundary conditions. The symmetric *q*-derivative operator was initially introduced in the domain of geometric function theory by Kanas et al. [31]. Khan et al. [32] have revised the generalized symmetric conic domain through the implementation of symmetric *q*-calculus concepts. In the study conducted by Khan et al. [33], a new category of *q*-starlike functions was introduced. The researchers also produced more findings about the symmetric *q*-operator, various *q*-starlike and *q*-convex functions, and techniques for extending the conic domain. A method was established by Khan et al. [34] to examine *m*-fold symmetric functions linked to *q*-symmetric calculus. This operator enabled the researchers to investigate specific significant results pertaining to *m*-fold symmetric bi-univalent functions. Khan et al. [35] further developed the notion of a symmetric *q*-derivative operator for multivalent functions and investigated various novel applications of this operator.

In this discussion, we have explored fundamental mathematical principles of symmetric *q*-calculus, which are crucial for understanding the topic presented in this article.

Al-Shbeil defined the symmetric *q*-difference operator ${\overset{ˆ}{D}}_{q}h(\tau )$ for h∈M using the same manner as the preceding definition.

Definition 2 [36]. The symmetric *q*-difference operator ${\overset{ˆ}{D}}_{q}h(\tau )$ for h∈M is defined by

$${\overset{ˆ}{D}}_{q}h(\tau )=\frac{h(q^{-1}\tau )-h(q\tau )}{\tau \left(q^{-1}-q\right)},\;\;\tau \in E^{⁎},=\frac{-1}{\tau^{2}}+\sum_{t=1}^{\infty }\overset{ˆ}{{\left[t\right]}_{q}}a_{t}\tau^{t-1},\;\;\left(\tau \neq 0,\;q\neq 1\right),$$

where $\overset{ˆ}{{\left[t\right]}_{q}}$ is defined in (6). It is evident that

$$\lim_{q\to 1-}{\overset{ˆ}{D}}_{q}F(\tau )=h^{{}^{\prime }}(\tau ),$$

and

$${\overset{ˆ}{D}}_{q}\tau^{t}=\overset{ˆ}{{\left[t\right]}_{q}}\tau^{m-1},\;\;\;{\overset{ˆ}{D}}_{q}\left\{\sum_{t=1}^{\infty }a_{t}\tau^{t}\right\}=\sum_{t=1}^{\infty }\overset{ˆ}{{\left[t\right]}_{q}}a_{t}\tau^{t-1}.$$

It is evident that

$$\lim_{q\to 1-}{\overset{ˆ}{D}}_{q}h(\tau )=h^{{}^{\prime }}(\tau ).$$

The expression $h^{{}^{\prime }}(\tau )$ represents the ordinary derivative, where as the term $\overset{ˆ}{{\left[t\right]}_{q}}$ is defined by

(6) $$\overset{ˆ}{{\left[t\right]}_{q}}=\frac{q^{-t}-q^{t}}{q^{-1}-q}.$$

The symmetric *q*-factorial ${\left[t\right]}_{q}!$ defined by:

$${\overset{ˆ}{\left[t\right]}}_{q}!=\prod_{k=1}^{t}\overset{ˆ}{{\left[k\right]}_{q}},\;\text{if}\;\;(t\in N)=1\;\;\text{if}\;\;t=0.$$

Remark 1 It is critical to recognize that a symmetric *q*-number cannot be reduced to *q*-number.

Remark 2 The potentially useful properties hold for F∈M

$$h(\tau )=h(\tau )⁎\frac{1}{\tau \left(1-\tau \right)}$$

and

$$h(\tau )=h(\tau )⁎\frac{1-{\overset{ˆ}{\left[2\right]}}_{q}}{\tau \left(1-q\tau \right)\left(1-q^{-1}\tau \right)}=-q^{2}\tau {\overset{ˆ}{D}}_{q}h(\tau ).$$

Let us now define a function ${\overset{ˆ}{R}}_{q}$ that has the following structure:

$${\overset{ˆ}{R}}_{q}(\tau )=\frac{1}{\tau }-\frac{\tau }{\left(1-q^{-1}\tau \right)\left(1-q\tau \right)}=\frac{1}{\tau }-\sum_{t=1}^{\infty }\overset{ˆ}{{\left[t\right]}_{q}}\tau^{t},$$

where $\overset{ˆ}{{\left[t\right]}_{q}}$ is defined in (6). Furthermore, it is evident that as q→1−, the value of ${\overset{ˆ}{R}}_{q}$ decreases in the following manner:

$$\lim_{q\to 1-}{\overset{ˆ}{R}}_{q}(\tau )=\frac{1}{\tau }-\frac{\tau }{{\left(1-\tau \right)}^{2}}=\frac{1}{\tau }-\sum_{t=1}^{\infty }t\tau^{t},\;\tau \in E^{⁎}.$$

Now, we develop the generalized symmetric quantum convolution derivative operator ${\overset{ˆ}{\mathrm{RD}}}_{q}$ for F∈M by using the function ${\overset{ˆ}{R}}_{q}$.

Definition 3 For F∈M, the generalized symmetric convolution *q*-derivative operator $\overset{ˆ}{\mathrm{RD}}$ is defined as follows:

(7) $${\overset{ˆ}{\mathrm{RD}}}_{q}h(\tau )=-\frac{1}{\tau }\left\{h(\tau )⁎\left(\frac{1}{\tau }-\frac{\tau }{\left(1-q\tau \right)\left(1-q^{-1}\tau \right)}\right)\right\}=-\frac{1}{\tau }\left\{h(\tau )⁎{\overset{ˆ}{R}}_{q}(\tau )\right\}=\frac{-1}{\tau^{2}}+\sum_{t=1}^{\infty }\overset{ˆ}{{\left[t\right]}_{q}}a_{t}\tau^{t-1},$$

where $\overset{ˆ}{{\left[t\right]}_{q}}$ is defined in (6).

Remark 3 For q=1, then we have $h^{{}^{\prime }}(\tau )=\frac{-1}{\tau^{2}}+\sum_{t=1}^{\infty }ta_{t}\tau^{t-1}$ and also, for 0<q<1, we obtain ${\overset{ˆ}{\mathrm{RD}}}_{q}h(\tau )={\overset{ˆ}{D}}_{q}h(\tau )$ defined in Definitions 2.

Right now, we use the generalized symmetric convolution *q*-derivative operator ${\overset{ˆ}{\mathrm{RD}}}_{q}$. Subsequently, we proceed to construct an operator for the function *F* that belongs to the set $M_{H}$.

Definition 4 Let $\overset{ˆ}{D_{H}^{q,v}}$:$M_{H}\to M_{H}$ is a new operator for a function $F=h+\bar{g}\in M_{H}$ by

(8) $$\overset{ˆ}{D_{H}^{q,v}}F(\tau )=\tau {\overset{ˆ}{\mathrm{RD}}}_{q}h(\tau )+v\bar{\tau {\overset{ˆ}{\mathrm{RD}}}_{q}g(\tau )},\;\;\left|v\right|=1.$$

In this paper, by using the $\overset{ˆ}{D_{H}^{q,v}}$ operator for meromorphic harmonic functions *F*, we define two new families, $MS_{H}^{P,R}\left(q,q^{-1}\right)$ and $MS_{\bar{H}}^{P,R}\left(q,q^{-1}\right)$ of the Janowski type functions, which are defined below:

Definition 5 Associated with $\overset{ˆ}{D_{H}^{q,v}}$:$M_{H}\to M_{H}$, we define a new class $MS_{H}^{P,R}\left(q,q^{-1}\right)$ of harmonic functions F∈H that satisfy following condition

(9) $$-\frac{\overset{ˆ}{D_{H}^{q,v}}F(\tau )}{F(\tau )}≺\frac{1+P\tau }{1+R\tau },\;q\in \left(0,1\right),\;\tau \in E^{⁎},-1\leq R<P\leq 1.$$

Inequality (9) is equivalent to the condition

(10) $$\left|\frac{\overset{ˆ}{D_{H}^{q,v}}F(\tau )+F(\tau )}{R\left(\overset{ˆ}{D_{H}^{q,v}}F(\tau )\right)+PF(\tau )}\right|<1.$$

Definition 6 To further elaborate, we represent the subclass $MS_{\bar{H}}^{P,R}\left(q,q^{-1}\right)$ consisting of harmonic meromorphic functions of the form (4) defined by the same way of (9) and (10).

## 2 Main results

### 2.1 Results for the function *F* in the class $MS_{H}^{P,R}\left(q,q^{-1}\right)$

**Necessary and sufficient condition**

Theorem 1 *For* −1≤R<P≤1 *,* 0<q<1,|v|=1 *. A function F given in* (2) *is in the class* $F\in MS_{H}^{P,R}\left(q,q^{-1}\right)$ *if and only if*

(11) $$0\neq \tau \{F(\tau )⁎(\frac{\left(1-\tau \right)\left(T\left(q,q^{-1}\right)-\tau^{2}\right)\left(1+R\zeta \right)+\left(1+Q\zeta \right)T\left(q,q^{-1}\right)}{\tau T\left(q,q^{-1}\right)\left(1-\tau \right)}+\frac{\left(\bar{T}\left(q,q^{-1}\right)-{\bar{\tau }}^{2}\right)\left(1-\bar{\tau }\right)\left(1+R\zeta \right)+\left(1+Q\zeta \right)\bar{T}\left(q,q^{-1}\right)}{\bar{\tau }\bar{T}\left(q,q^{-1}\right)\left(1-\bar{\tau }\right)})\},$$

*where*

(12) $$T\left(q,q^{-1}\right)=\left(1-q\tau \right)\left(1-q^{-1}\tau \right)\;\text{and}\;\bar{T}\left(q,q^{-1}\right)=\left(1-q\bar{\tau }\right)\left(1-q^{-1}\bar{\tau }\right).$$

Proof Let $F=h+\bar{g}$ be of the form (2) and $F\in MS_{H}^{P,R}\left(q,q^{-1}\right)$. In accordance with the principle of weak subordination, there is a function *u* that satisfies the constraints u(∞)=∞ and |u(τ)|<1, such that

(13) $$-\frac{\overset{ˆ}{D_{H}^{q,v}}F(\tau )}{F(\tau )}=\frac{1+Pu(\tau )}{1+Ru(\tau )},$$

which is equal to

(14) $$-\frac{\overset{ˆ}{D_{H}^{q,v}}F(\tau )}{F(\tau )}\neq \frac{1+P\zeta }{1+R\zeta },\;\;\left(\zeta \in C,\;\left|\zeta \right|=1\right)$$

or

(15) $$\tau \left[-\overset{ˆ}{D_{H}^{q,v}}F(\tau )\left(1+R\zeta \right)-F(\tau )\left(1+P\zeta \right)\right]\neq 0.$$

Since (8) we have

$$\overset{ˆ}{D_{H}^{q,v}}F(\tau )=\tau {\overset{ˆ}{\mathrm{RD}}}_{q}h(\tau )+v\bar{\tau {\overset{ˆ}{\mathrm{RD}}}_{q}g(\tau )}=\left\{h(\tau )⁎\frac{T\left(q,q^{-1}\right)-\tau^{2}}{\tau T\left(q,q^{-1}\right)}\right\}+v\left\{\bar{g(\tau )}⁎\frac{\bar{T}\left(q,q^{-1}\right)-{\bar{\tau }}^{2}}{\bar{\tau }\bar{T}\left(q,q^{-1}\right)}\right\},$$

together with following identities:

$$h(\tau )⁎\frac{1}{\tau \left(1-\tau \right)}=h(\tau )$$

and

$$\bar{g(\tau )}⁎\frac{1}{\bar{\tau }\left(1-\bar{\tau }\right)}=\bar{g(\tau )},$$

where $T\left(q,q^{-1}\right)$ and $\bar{T}\left(q,q^{-1}\right)$ is given by (12). We find from (15) that

$$0\neq \tau \left[-\overset{ˆ}{D_{H}^{q,v}}F(\tau )\left(1+R\zeta \right)-F(\tau )\left(1+P\zeta \right)\right],0\neq \tau (-\left(\left\{h(\tau )⁎\frac{T\left(q,q^{-1}\right)-\tau^{2}}{\tau T\left(q,q^{-1}\right)}\right\}+v\left\{\bar{g(\tau )}⁎\frac{\bar{T}\left(q,q^{-1}\right)-{\bar{\tau }}^{2}}{\bar{\tau }\bar{T}\left(q,q^{-1}\right)}\right\}\right)\left(1+R\zeta \right)-\left(h(\tau )⁎\frac{1}{\tau \left(1-\tau \right)}+v\bar{g(\tau )}⁎\frac{1}{\bar{\tau }\left(1-\bar{\tau }\right)}\right)\left(1+P\zeta \right)),\neq \tau (-\left(h(\tau )⁎\frac{T\left(q,q^{-1}\right)-\tau^{2}}{\tau T\left(q,q^{-1}\right)}\right)\left(1+R\zeta \right)-v\left(\bar{g(\tau )}⁎\frac{\bar{T}\left(q,q^{-1}\right)-{\bar{\tau }}^{2}}{\bar{\tau }\bar{T}\left(q,q^{-1}\right)}\right)\left(1+R\zeta \right)-\left(h(\tau )⁎\frac{1}{\tau \left(1-\tau \right)}\right)\left(1+P\zeta \right)-v\left(\bar{g(\tau )}⁎\frac{1}{\bar{\tau }\left(1-\bar{\tau }\right)}\right)\left(1+P\zeta \right))\neq \tau (-\left(\left(h(\tau )⁎\frac{T\left(q,q^{-1}\right)-\tau^{2}}{\tau T\left(q,q^{-1}\right)}\right)\left(1+R\zeta \right)+\left(h(\tau )⁎\frac{1}{\tau \left(1-\tau \right)}\right)\left(1+P\zeta \right)\right)-v\left(\left(\bar{g(\tau )}⁎\frac{\bar{T}\left(q,q^{-1}\right)-{\bar{\tau }}^{2}}{\bar{\tau }\bar{T}\left(q,q^{-1}\right)}\right)\left(1+R\zeta \right)+\left(\bar{g(\tau )}⁎\frac{1}{\bar{\tau }\left(1-\bar{\tau }\right)}\right)\left(1+P\zeta \right)\right)),\neq -\tau ((h(\tau )⁎\left(\frac{\left(1-\tau \right)\left(T\left(q,q^{-1}\right)-\tau^{2}\right)\left(1+R\zeta \right)+\left(1+P\zeta \right)T\left(q,q^{-1}\right)}{\tau T\left(q,q^{-1}\right)\left(1-\tau \right)}\right)+v\bar{g(\tau )}⁎\left(\frac{\left(\left(1-q\bar{\tau }\right)\left(1-q^{-1}\bar{\tau }\right)-{\bar{\tau }}^{2}\right)\left(1-\bar{\tau }\right)\left(1+R\zeta \right)+\left(1+P\zeta \right)\left(1-q\bar{\tau }\right)\left(1-q^{-1}\bar{\tau }\right)}{\bar{\tau }\left(1-q\bar{\tau }\right)\left(1-q^{-1}\bar{\tau }\right)\left(1-\bar{\tau }\right)}\right))),$$

$$0\neq \tau \{-F(\tau )⁎(\frac{\left(1-\tau \right)\left(T\left(q,q^{-1}\right)-\tau^{2}\right)\left(1+R\zeta \right)+\left(1+P\zeta \right)T\left(q,q^{-1}\right)}{\tau T\left(q,q^{-1}\right)\left(1-\tau \right)}+\frac{\left(\bar{T}\left(q,q^{-1}\right)-{\bar{\tau }}^{2}\right)\left(1-\bar{\tau }\right)\left(1+R\zeta \right)+\left(1+P\zeta \right)\bar{T}\left(q,q^{-1}\right)}{\bar{\tau }\bar{T}\left(q,q^{-1}\right)\left(1-\bar{\tau }\right)})\}.$$

This finally results in (11), and the essential component of the proof of Theorem 1 has been finished.

Conversely: On the other hand, if we assume that (11) is correct, then it follows that −τF(τ)0≠0 is not equal to zero for any *τ* that belongs to the set $E^{⁎}$. Since this is the case, the function ϕ(τ) by

$$\phi \left(\tau \right)=-\frac{\overset{ˆ}{D_{H}^{q,v}}F(\tau )}{F(\tau )}$$

is analytic at $\tau_{0}=0$, with ϕ(0)=1. The assumption (11) was shown to be identical to (14), which means that

(16) $$-\frac{\overset{ˆ}{D_{H}^{q,v}}F(\tau )}{F(\tau )}\neq \frac{1+P\zeta }{1+R\zeta },\;\left(\zeta \in C,\;\left|\zeta \right|=1\right).$$

Let

$$\Psi (\tau )=\frac{1+P\zeta }{1+R\zeta },\;\tau \in E.$$

Then the relation (16) demonstrates that ϕ(E)∩Ψ(∂E)=0. As a result, we see that ϕ(0)=Ψ(0) which, is in the connection with the univalence of Ψ, demonstrates that ϕ(τ)≤Ψ(τ) in sense of the definition of subordination in (13), that is $F\in MS_{H}^{P,R}\left(q,q^{-1}\right)$. This concludes the proof of Theorem 1.

Theorem 2 *A function F given in* (2) *belongs to the class* $MS_{H}^{P,R}\left(q,q^{-1}\right)$ *if and only if*

$$1+\sum_{t=1}^{\infty }\frac{\left(\zeta^{-1}+R\right)\overset{ˆ}{{\left[t\right]}_{q}}+\zeta^{-1}+P}{P-R}\left(a_{t}\tau^{t+1}+v\bar{b_{t}\tau^{t+1}}\right)\neq 0.$$

Proof By Theorem 1, we know the fact that $F\in MS_{H}^{P,R}\left(q,q^{-1}\right)$ if and only if

(17) $$\tau \{F(\tau )⁎(\frac{\left(1-\tau \right)\left(T\left(q,q^{-1}\right)-\tau^{2}\right)\left(1+R\zeta \right)+\left(1+P\zeta \right)T\left(q,q^{-1}\right)}{\tau T\left(q,q^{-1}\right)\left(1-\tau \right)}+\frac{\left(\bar{T}\left(q,q^{-1}\right)-{\bar{\tau }}^{2}\right)\left(1-\bar{\tau }\right)\left(1+R\zeta \right)+\left(1+P\zeta \right)\bar{T}\left(q,q^{-1}\right)}{\bar{\tau }\bar{T}\left(q,q^{-1}\right)\left(1-\bar{\tau }\right)})\}\neq 0.$$

Taking the left-hand side of (17) and then can be written as:

$$\tau \{h(\tau )⁎(\frac{\left(1-\tau \right)\left(T\left(q,q^{-1}\right)-\tau^{2}\right)\left(1+R\zeta \right)+\left(1+P\zeta \right)T\left(q,q^{-1}\right)}{\tau T\left(q,q^{-1}\right)\left(1-\tau \right)}+v\bar{g(\tau )}⁎\frac{\left(\bar{T}\left(q,q^{-1}\right)-{\bar{\tau }}^{2}\right)\left(1-\bar{\tau }\right)\left(1+R\zeta \right)+\left(1+P\zeta \right)\bar{T}\left(q,q^{-1}\right)}{\bar{\tau }\bar{T}\left(q,q^{-1}\right)\left(1-\bar{\tau }\right)})\},$$

$$\tau \{h(\tau )⁎(\frac{\left(1+R\zeta \right)}{\tau }-\frac{\tau \left(1+R\zeta \right)}{T\left(q,q^{-1}\right)}+\frac{\left(1+P\zeta \right)}{\tau \left(1-\tau \right)}+v\bar{g(\tau )}⁎\left(\frac{\left(1+R\zeta \right)}{\bar{\tau }}-\frac{\bar{\tau }\left(1+R\zeta \right)}{\bar{T}\left(q,q^{-1}\right)}+\frac{\left(1+P\zeta \right)}{\bar{\tau }\left(1-\bar{\tau }\right)}\right))\},$$

and

$$\tau \{h(\tau )⁎(\left(1+R\zeta \right)\left(\frac{1}{\tau }-\frac{\tau }{T\left(q,q^{-1}\right)}\right)+\left(1+P\zeta \right)\frac{1}{\tau \left(1-\tau \right)}+v\bar{g(\tau )}⁎\left(\left(1+R\zeta \right)\left(\frac{1}{\bar{\tau }}-\frac{\bar{\tau }}{\bar{T}\left(q,q^{-1}\right)}\right)+\left(1+P\zeta \right)\frac{1}{\bar{\tau }\left(1-\bar{\tau }\right)}\right)\}.$$

Following a few straightforward computations, we obtain

$$\{\left(P-R\right)\zeta +\sum_{t=1}^{\infty }\left(\left(1+R\zeta \right)\overset{ˆ}{{\left[t\right]}_{q}}+\left(1+P\zeta \right)\right)a_{t}\tau^{t+1}+v\left(\left(P-R\right)\zeta +\sum_{t=1}^{\infty }\left(\left(1+R\zeta \right)\overset{ˆ}{{\left[t\right]}_{q}}+\left(1+P\zeta \right)\right)\bar{b_{t}\tau^{t+1}}\right)\}.$$

With this, it is clear that our proof of the conclusion that is declared by Theorem 2 has been completed.

### 2.2 Results for the *F* in the class $MS_{\bar{H}}^{P,R}\left(q,q^{-1}\right)$

Theorem 3 *A function F defined by* (4) *. If*

(18) $$\sum_{t=1}^{\infty }\frac{\left|\left(1-R\right){\overset{ˆ}{\left[t\right]}}_{q}+\left(1-P\right)\right|}{\left|P-R\right|}\left(\left|a_{t}\right|+\left|b_{t}\right|\right)\leq 1.$$

*Then (i) the function F is univalent and sense-preserving as* q→1− *in* $E^{⁎}$ *and (ii) the function* $F\in MS_{\bar{H}}^{P,R}\left(q,q^{-1}\right)$ *.*

Proof For $0<\left|\tau_{1}\right|\leq \left|\tau_{2}\right|<1$, we have

$$\left|\frac{F(\tau_{1})-F(\tau_{2})}{h(\tau_{1})-h(\tau_{2})}\right|\geq 1-\left|\frac{g(\tau_{1})-g(\tau_{2})}{h(\tau_{1})-h(\tau_{2})}\right|=1-\left|\frac{\tau_{1}\tau_{2}\sum_{t=1}^{\infty }b_{t}\left(\tau_{1}^{t}-\tau_{2}^{t}\right)}{\left(\tau_{1}-\tau_{2}\right)-\tau_{1}\tau_{2}\sum_{t=1}^{\infty }a_{t}\left(\tau_{1}^{t}-\tau_{2}^{t}\right)}\right|>1-\frac{\sum_{t=1}^{\infty }tb_{t}}{1-\sum_{t=1}^{\infty }ta_{t}}>1-\frac{\sum_{t=1}^{\infty }\frac{\left|\left(1-R\right){\overset{ˆ}{\left[t\right]}}_{q}+\left(1-P\right)\right|}{\left|P-R\right|}b_{t}}{1-\sum_{t=1}^{\infty }\frac{\left|\left(1-R\right){\overset{ˆ}{\left[t\right]}}_{q}+\left(1-P\right)\right|}{\left|P-R\right|}a_{t}}\geq 0.$$

This demonstrates that the function is univalent.

Now, let's demonstrate that F(τ) is a sense-preserving harmonic mapping in the domain $E^{⁎}$. Let

$$\left|{\overset{ˆ}{D}}_{q}h(\tau )\right|\geq \frac{1}{{\left|\tau \right|}^{2}}-\sum_{t=1}^{\infty }{\overset{ˆ}{\left[t\right]}}_{q}\left|a_{t}\right|{\left|\tau \right|}^{t-1}\geq 1-\sum_{t=1}^{\infty }\frac{\left|\left(1-R\right){\overset{ˆ}{\left[t\right]}}_{q}+\left(1-P\right)\right|}{\left|P-R\right|}\left|a_{t}\right|\geq \sum_{t=1}^{\infty }\frac{\left|\left(1-R\right){\overset{ˆ}{\left[t\right]}}_{q}+\left(1-P\right)\right|}{\left|P-R\right|}\left|b_{t}\right|\geq \sum_{t=1}^{\infty }{\overset{ˆ}{\left[t\right]}}_{q}\left|b_{t}\right|\geq {\overset{ˆ}{D}}_{q}g(\tau ).$$

Applying that q→1− that $\left|h^{{}^{\prime }}(\tau )\right|>\left|g^{{}^{\prime }}(\tau )\right|$ in $E^{⁎}$ indicating that *F* is sense-preserving.

To prove that $F\in MS_{\bar{H}}^{P,R}\left(q,q^{-1}\right)$, it is enough to show that

(19) $$\left|\overset{ˆ}{D_{H}^{q,v}}F(\tau )+F(\tau )\right|-\left|R\left(\overset{ˆ}{D_{H}^{q,v}}F(\tau )\right)+PF(\tau )\right|<0.$$

Consider

$$\left|\overset{ˆ}{D_{H}^{q,v}}F(\tau )+F(\tau )\right|-\left|R\left(\overset{ˆ}{D_{H}^{q,v}}F(\tau )\right)+PF(\tau )\right|=\left|\sum_{t=1}^{\infty }\left({\overset{ˆ}{\left[t\right]}}_{q}+1\right)a_{t}\tau^{t}+\bar{v\sum_{t=1}^{\infty }\left({\overset{ˆ}{\left[t\right]}}_{q}+1\right)b_{t}\tau^{t}}\right|-\left|\frac{P-R}{\tau }+\sum_{t=1}^{\infty }\left(R{\overset{ˆ}{\left[t\right]}}_{q}+P\right)a_{t}\tau^{t}+v\sum_{t=1}^{\infty }\bar{\left(R{\overset{ˆ}{\left[t\right]}}_{q}+P\right)b_{t}\tau^{t}}\right|-\leq \sum_{t=1}^{\infty }\left({\overset{ˆ}{\left[t\right]}}_{q}+1\right)\left|a_{t}\right|{\left|\tau \right|}^{t}+\sum_{t=1}^{\infty }\left({\overset{ˆ}{\left[t\right]}}_{q}+1\right)\left|b_{t}\right|\left|\tau^{t}\right|-\left|\frac{P-R}{\tau }\right|-\sum_{t=1}^{\infty }\left(R{\overset{ˆ}{\left[t\right]}}_{q}+P\right)\left|a_{t}\right|{\left|\tau \right|}^{t}-\sum_{t=1}^{\infty }\left(R{\overset{ˆ}{\left[t\right]}}_{q}+P\right)\left|b_{t}\right|{\bar{\left|\tau \right|}}^{t}=\sum_{t=1}^{\infty }\left(\left(1-R\right){\overset{ˆ}{\left[t\right]}}_{q}+\left(1+P\right)\right)\left|a_{t}\right|{\left|\tau \right|}^{t}+\sum_{t=1}^{\infty }\left(\left(1-R\right){\overset{ˆ}{\left[t\right]}}_{q}-\left(1-P\right)\right)\left|b_{t}\right|{\bar{\left|\tau \right|}}^{t}-\frac{\left|P-R\right|}{\left|\tau \right|}=\sum_{t=1}^{\infty }\left(\left(1-R\right){\overset{ˆ}{\left[t\right]}}_{q}+\left(1-P\right)\right)\left(\left|a_{t}\right|+\left|b_{t}\right|\right)-\left|P-R\right|.$$

By (19), we get

$$\sum_{t=1}^{\infty }\left(\left(1-R\right){\overset{ˆ}{\left[t\right]}}_{q}+\left(1-P\right)\right)\left(\left|a_{t}\right|+\left|b_{t}\right|\right)-\left|P-R\right|<0.$$

Hence

$$\sum_{t=1}^{\infty }\frac{\left(\left(1-R\right){\overset{ˆ}{\left[t\right]}}_{q}+\left(1-P\right)\right)}{\left|P-R\right|}\left(\left|a_{t}\right|+\left|b_{t}\right|\right)<1.$$

Example 1 The meromorphic univalent function

$$F(\tau )=\frac{1}{\tau }+\sum_{t=1}^{\infty }\frac{\left|P-R\right|X_{t}}{\left(1-R\right){\overset{ˆ}{\left[t\right]}}_{q}+\left(1-P\right)}\tau^{t}+\sum_{t=1}^{\infty }\frac{\left|P-R\right|Y_{t}}{\left(1-R\right){\overset{ˆ}{\left[t\right]}}_{q}+\left(1-P\right)}\bar{\tau^{t}},$$

such that

$$\sum_{t=1}^{\infty }X_{t}\tau^{t}+\sum_{t=1}^{\infty }Y_{t}\bar{\tau^{t}}=1,$$

we have

$$\sum_{t=1}^{\infty }\frac{\left(1-R\right){\overset{ˆ}{\left[t\right]}}_{q}+\left(1-P\right)}{\left|P-R\right|}\left(\left|a_{t}\right|+\left|b_{t}\right|\right)=\sum_{t=1}^{\infty }\left(\frac{\left(1-R\right){\overset{ˆ}{\left[t\right]}}_{q}+\left(1-P\right)}{\left|P-R\right|}\left|a_{t}\right|+\frac{\left(1-R\right){\overset{ˆ}{\left[t\right]}}_{q}+\left(1-P\right)}{\left|P-R\right|}\left|b_{t}\right|\right)=\sum_{t=1}^{\infty }\left(\frac{\left(1-R\right){\overset{ˆ}{\left[t\right]}}_{q}+\left(1-P\right)}{\left|P-R\right|}\left|a_{t}\right|+\frac{\left(1-R\right){\overset{ˆ}{\left[t\right]}}_{q}+\left(1-P\right)}{\left|P-R\right|}\left|b_{t}\right|\right)=\sum_{t=1}^{\infty }\left(\left|X_{t}\right|+\left|Y_{t}\right|\right)=1.$$

Thus $F\in MS\bar{{}_{H}}\left(P,R,q,q^{-1}\right)$ and above coefficient bound given in (18) is sharp for this function.

Theorem 4 *A function F defined by* (4) *then* $F\in MS_{\bar{H}}^{P,R}\left(q,q^{-1}\right)$ *if*

$$\sum_{t=1}^{\infty }\frac{\left|\left(1-R\right){\overset{ˆ}{\left[t\right]}}_{q}+\left(1-P\right)\right|}{\left|P-R\right|}\left(\left|a_{t}\right|+\left|b_{t}\right|\right)\leq 1.$$

Proof The proof is excluded because it proceeds along lines that are similar to Theorem 2.

Theorem 5 *Let* $F=h+\bar{g}$ *defined by* (4) *is in the class* $MS_{\bar{H}}^{P,R}\left(q,q^{-1}\right)$ *,* 0<|τ|=r<1 *. Then*

$$\frac{1}{r}-\frac{\left|P-R\right|}{2-\left(R+P\right)}r\leq \left|F(\tau )\right|\leq \frac{1}{r}+\frac{\left|P-R\right|}{2-\left(R+P\right)}r.$$

Proof Consider

$$\left|F(\tau )\right|=\left|\frac{1}{\tau }+\sum_{t=1}^{\infty }\left(a_{t}\tau^{t}+b_{t}\bar{\tau }\right)\right|\leq \frac{1}{r}+\sum_{t=1}^{\infty }\left(\left|a_{t}\right|+\left|b_{t}\right|\right)r^{t}\leq \frac{1}{r}+\sum_{t=1}^{\infty }\left(\left|a_{t}\right|+\left|b_{t}\right|\right)r\leq \frac{1}{r}+\frac{\left|P-R\right|}{2-\left(R+P\right)}r.$$

Similarly

$$\left|F(\tau )\right|=\left|\frac{1}{\tau }+\sum_{t=1}^{\infty }\left(a_{t}\tau^{t}+b_{t}\bar{\tau }\right)\right|\geq \frac{1}{r}-\sum_{t=1}^{\infty }\left(\left|a_{t}\right|+\left|b_{t}\right|\right)r^{t}\geq \frac{1}{r}-\sum_{t=1}^{\infty }\left(\left|a_{t}\right|+\left|b_{t}\right|\right)r\geq \frac{1}{r}-\frac{\left|P-R\right|}{2-\left(R+P\right)}r.$$

Hence, this completes the result.

Theorem 6 *Let* $F=h+\bar{g}$ *defined by* (4) *is in the class* $MS_{\bar{H}}^{P,R}\left(q,q^{-1}\right)$ *, if and only if*

$$F(\tau )=\sum_{t=0}^{\infty }\left(A_{t}h_{t}+B_{t}g_{t}\right)$$

*where*

$$h_{t}(\tau )=\frac{1}{\tau }+\frac{P-R}{\left|\left(1-R\right){\overset{ˆ}{\left[t\right]}}_{q}+\left(1-P\right)\right|}\tau^{t},\;\;t\geq 1,g_{t}(\tau )=\frac{1}{\tau }-\frac{P-R}{\left|\left(1-R\right){\overset{ˆ}{\left[t\right]}}_{q}+\left(1-P\right)\right|}\bar{\tau^{t}},\;\;t\geq 1$$

$$h_{0}(\tau )=\frac{1}{\tau },g_{0}(\tau )=\frac{1}{\bar{\tau }}$$

*and*

$$1\geq A_{t}\geq 0,\;1\geq B_{t}\geq 0,\;\sum_{t=0}^{\infty }\left(A_{t}+B_{t}\right)=1.$$

Proof Let

$$F(\tau )=A_{0}h_{0}+B_{0}g_{0}+F(\tau )+\sum_{t=1}^{\infty }\left(A_{t}h_{t}+B_{t}g_{t}\right)=\left(A_{0}+B_{0}\right)\frac{1}{\tau }+\sum_{t=1}^{\infty }A_{t}\left(\frac{1}{\tau }+\frac{P-R}{\left|\left(1-R\right){\overset{ˆ}{\left[t\right]}}_{q}+\left(1-P\right)\right|}\tau^{t}\right)+\sum_{t=1}^{\infty }B_{t}\left(\frac{1}{\tau }-\frac{P-R}{\left|\left(1-R\right){\overset{ˆ}{\left[t\right]}}_{q}+\left(1-P\right)\right|}\bar{\tau^{t}}\right)$$

$$=\left(A_{0}+B_{0}\right)\frac{1}{\tau }+\sum_{t=1}^{\infty }\left(A_{t}+B_{t}\right)+\sum_{t=1}^{\infty }A_{t}\frac{P-R}{\left|\left(1-R\right){\overset{ˆ}{\left[t\right]}}_{q}+\left(1-P\right)\right|}\tau^{t}-\sum_{t=1}^{\infty }B_{t}\frac{P-R}{\left|\left(1-R\right){\overset{ˆ}{\left[t\right]}}_{q}+\left(1-P\right)\right|}\bar{\tau^{t}}.$$

Thus,

$$\sum_{t=1}^{\infty }\frac{\left|\left(1-R\right){\overset{ˆ}{\left[t\right]}}_{q}+\left(1-P\right)\right|}{\left|P-R\right|}\left(\frac{\left|P-R\right|A_{t}}{\left|\left(1-R\right){\overset{ˆ}{\left[t\right]}}_{q}+\left(1-P\right)\right|}+\frac{\left|P-R\right|B_{t}}{\left|\left(1-R\right){\overset{ˆ}{\left[t\right]}}_{q}+\left(1-P\right)\right|}\right)$$

$$=\sum_{t=1}^{\infty }\left(A_{t}+B_{t}\right)=1-A_{0}-B_{0}\leq 1.$$

Hence $F\in MS_{\bar{H}}^{P,R}\left(q,q^{-1}\right)$. Conversely, let $F\in MS_{\bar{H}}^{P,R}\left(q,q^{-1}\right)$. Set

$$A_{t}=\frac{\left|\left(1-R\right){\overset{ˆ}{\left[t\right]}}_{q}+\left(1-P\right)\right|}{\left|P-R\right|}\left|a_{t}\right|,\;1\geq A_{t}\geq 0,B_{t}=\frac{\left|\left(1-R\right){\overset{ˆ}{\left[t\right]}}_{q}+\left(1-P\right)\right|}{\left|P-R\right|}\left|b_{t}\right|,\;\;1\geq B_{t}\geq 0.$$

Therefore, *F* can be written as:

$$F(\tau )=\frac{1}{\tau }+\sum_{t=1}^{\infty }\left|a_{t}\right|\tau^{t}-\sum_{t=1}^{\infty }\left|b_{t}\right|\bar{\tau^{t}}=\frac{1}{\tau }+\sum_{t=1}^{\infty }\frac{\left|P-R\right|A_{t}}{\left|\left(1-R\right){\overset{ˆ}{\left[t\right]}}_{q}+\left(1-P\right)\right|}\tau^{t}-\sum_{t=1}^{\infty }\frac{\left|P-R\right|B_{t}}{\left|\left(1-R\right){\overset{ˆ}{\left[t\right]}}_{q}+\left(1-P\right)\right|}\bar{\tau^{t}},$$

$$=\left(A_{0}+B_{0}\right)\frac{1}{\tau }+\sum_{t=1}^{\infty }\left(\frac{1}{\tau }+\frac{\left|P-R\right|}{\left|\left(1-R\right){\overset{ˆ}{\left[t\right]}}_{q}+\left(1-P\right)\right|}\tau^{t}\right)A_{t}+\sum_{t=1}^{\infty }B_{t}\left(\frac{1}{\tau }-\frac{\left|P-R\right|}{\left|\left(1-R\right){\overset{ˆ}{\left[t\right]}}_{q}+\left(1-P\right)\right|}\bar{\tau^{t}}\right).$$

Hence

$$F(\tau )=\sum_{t=0}^{\infty }\left(A_{t}h_{t}+B_{t}g_{t}\right).$$

Theorem 7 *The class* $MS_{\bar{H}}^{P,R}\left(q,q^{-1}\right)$ *is closed under convex combination.*

Proof Let $F_{k}\in MS_{\bar{H}}^{P,R}\left(q,q^{-1}\right)$ be of the form

(20) $$F_{k}(\tau )=\frac{1}{\tau }+\sum_{t=1}^{\infty }\left|a_{k,t}\right|\tau^{t}-\sum_{t=1}^{\infty }\left|b_{k,t}\right|\bar{\tau^{t}},\;k\in N.$$

Then form (18), we get

$$\sum_{t=1}^{\infty }\frac{\left|\left(1-R\right){\overset{ˆ}{\left[t\right]}}_{q}+\left(1-P\right)\right|}{\left|P-R\right|}\left(\left|a_{k,t}\right|+\left|b_{k,t}\right|\right)\leq 1.$$

For $\sum_{t=1}^{\infty }\delta_{k}=1$, $\left(0\leq \delta_{k}\leq 1\right)$, then $F_{k}$ can be written in convex combination form

$$\sum_{k=1}^{\infty }\delta_{k}F_{k}(\tau )=\frac{1}{\tau }+\sum_{t=1}^{\infty }\left(\sum_{k=1}^{\infty }\delta_{k}\left|a_{k,t}\right|\right)\tau^{t}-\sum_{t=1}^{\infty }\left(\sum_{k=1}^{\infty }\delta_{k}\left|b_{k,t}\right|\right)\bar{\tau^{t}}.$$

Using (18), we have

$$\sum_{t=1}^{\infty }\frac{\left|\left(1-R\right){\overset{ˆ}{\left[t\right]}}_{q}+\left(1-P\right)\right|}{\left|P-R\right|}\left(\sum_{k=1}^{\infty }\delta_{k}\left|a_{k,t}\right|\right)\tau^{t}-\sum_{t=1}^{\infty }\left(\sum_{k=1}^{\infty }\delta_{k}\left|b_{k,t}\right|\right)\bar{\tau^{t}}=\sum_{k=1}^{\infty }\delta_{k}\left(\sum_{t=1}^{\infty }\frac{\left|\left(1-R\right){\overset{ˆ}{\left[t\right]}}_{q}+\left(1-P\right)\right|}{\left|P-R\right|}\left(\left|a_{k,t}\right|+\left|b_{k,t}\right|\right)\right)\leq \sum_{k=1}^{\infty }\delta_{k}=1,$$

thus prove our desired results.

Theorem 8 $F_{k}\in MS_{\bar{H}}^{P,R}\left(q,q^{-1}\right)$ *for* k={1,2} *be of the form* (20) *, then, their weighted mean* $F_{i}\in MS_{\bar{H}}^{P,R}\left(q,q^{-1}\right)$ *where* $F_{i}$ *is defined below*

(21) $$F_{i}\left(\tau \right)=\frac{\left(1-i\right)F_{1}(\tau )+(1+i)F_{2}(\tau )}{2}.$$

Proof It is simple to write from equation (21):

$$F_{i}\left(\tau \right)=\frac{\left(1-i\right)F_{1}(\tau )+(1+i)F_{2}(\tau )}{2}=\frac{1}{\tau }+\sum_{t=1}^{\infty }\{\left(\frac{\left(1-i\right)\left|a_{1,t}\right|+\left(1+i\right)\left|a_{2,t}\right|}{2}\right)\tau^{t}-\left(\frac{\left(1-i\right)\left|b_{1,t}\right|+\left(1+i\right)\left|b_{2,t}\right|}{2}\right)\bar{\tau^{t}}\}.$$

To show that $F_{i}\in MS_{\bar{H}}^{P,R}\left(q,q^{-1}\right)$ it is enough to show that

$$\sum_{t=1}^{\infty }\frac{\left|\left(1-R\right){\overset{ˆ}{\left[t\right]}}_{q}+\left(1-P\right)\right|}{\left|P-R\right|}\{\left|\frac{\left(1-i\right)\left|a_{1,t}\right|+\left(1+i\right)\left|a_{2,t}\right|}{2}\right|+\left|\frac{\left(1-i\right)\left|b_{1,t}\right|+\left(1+i\right)\left|b_{2,t}\right|}{2}\right|\}\leq 1.$$

Now consider

$$\sum_{t=1}^{\infty }\frac{\left|\left(1-R\right){\overset{ˆ}{\left[t\right]}}_{q}+\left(1-P\right)\right|}{\left|P-R\right|}\{\left|\frac{\left(1-i\right)\left|a_{1,t}\right|+\left(1+i\right)\left|a_{2,t}\right|}{2}\right|+\left|\frac{\left(1-i\right)\left|b_{1,t}\right|+\left(1+i\right)\left|b_{2,t}\right|}{2}\right|\}=\sum_{t=1}^{\infty }\frac{\left|\left(1-R\right){\overset{ˆ}{\left[t\right]}}_{q}+\left(1-P\right)\right|}{\left|P-R\right|}\{\left|\frac{\left(1-i\right)\left|a_{1,t}\right|+\left(1-i\right)\left|b_{1,t}\right|}{2}\right|+\left|\frac{\left(1+i\right)\left|a_{2,t}\right|+\left(1+i\right)\left|b_{2,t}\right|}{2}\right|\}$$

$$=\frac{\left(1-i\right)}{2}\sum_{t=1}^{\infty }\frac{\left|\left(1-R\right){\overset{ˆ}{\left[t\right]}}_{q}+\left(1-P\right)\right|}{\left|P-R\right|}\left(\left|a_{1,t}\right|+\left|b_{1,t}\right|\right)+\frac{\left(1+i\right)}{2}\sum_{t=1}^{\infty }\frac{\left|\left(1-R\right){\overset{ˆ}{\left[t\right]}}_{q}+\left(1-P\right)\right|}{\left|P-R\right|}\left(\left|a_{2,t}\right|+\left|b_{2,t}\right|\right)\leq \frac{\left(1-i\right)}{2}+\frac{\left(1+i\right)}{2}=1.$$

Hence, $F_{i}\in MS_{\bar{H}}^{P,R}\left(q,q^{-1}\right)$.

## 3 Conclusions

This article explores new applications of symmetric *q*-calculus in the context of meromorphic harmonic functions in $E^{⁎}$, leading to the discovery of new subclasses. We began by defining the *q*-analogues of the functions ${\overset{ˆ}{R}}_{q}$ and then utilized these functions, in conjunction with convolution concepts, to establish the *q*-analogous generalized symmetric convolution operator $D_{H}^{q,v}$ for meromorphic harmonic functions. Building on the operator $D_{H}^{q,v}$, we introduced new subclasses of meromorphic harmonic functions and explored their relationship with Janowski functions. Our investigation revealed key geometric features of functions in the class $MS_{H}^{P,R}\left(q,q^{-1}\right)$, including convolution properties, sufficiency conditions, distortion bounds, convex combinations, extreme point theorem, and weighted mean estimates, which provide valuable insights into the geometric behavior of these functions.

## Use of AI tools declaration

The authors declare they have not used Artificial Intelligence (AI) tools in the creation of this article.

## Funding

This work was funded through Arab Open University research fund no. (AOUKSA-524008).

## CRediT authorship contribution statement

**Ahmad A. Abubakar:** Writing – review & editing, Writing – original draft. **Khaled Matarneh:** Writing – review & editing. **Suha B. Al-Shaikh:** Writing – review & editing, Supervision, Funding acquisition. **Mohammad Faisal Khan:** Writing – review & editing. **Mustafa Kamal:** Writing – review & editing.

## Declaration of Competing Interest

The authors declare that they have no known competing financial interests or personal relationships that could have appeared to influence the work reported in this paper.

## Data Availability

No data was used for the research described in the article.
